# An integrated review of the role of communication in veterinary clinical practice

**DOI:** 10.1186/s12917-020-02558-2

**Published:** 2020-10-19

**Authors:** Jack K. H. PUN

**Affiliations:** Department of English, City University of Hong Kong, 83, Tat Chee Avenue, Kowloon, Hong Kong

**Keywords:** Veterinary medicine, Clinical communication, English-speaking countries, Clinical veterinary education

## Abstract

**Background:**

There is a growing interest in exploring the nature of communication in veterinary medicine and understanding how veterinary practitioners communicate with their clients and other professionals. This is the first integrative review of literature on veterinary communication. Applying the PRISMA model, the PubMed, PsychInfo and ERIC databases were searched using keywords such as ‘veterinary’, ‘vet’, ‘communication’ and ‘interaction’ for related articles published between 1 January 2000 and 31 December 2018.

**Results:**

Keyword searching through the databases yielded 1572 related studies. Only 48 of these studies were included in our analysis after an in-depth review by two independent reviewers using the critical appraisal skills Programme frameworks with high inter-rater reliability (Cohen’s kappa coefficient κ > 0.8). The existing body of research on veterinary communication can be classified into three major areas: (a) client–veterinarian communication, (b) cross-disciplinary communication in a professional veterinarian team and (c) training of veterinary communication skills. This review details the complexity and heterogeneity of agenda in the field of veterinary communication. The included studies indicate that veterinary practitioners are not equipped with specific communication skills to address different agendas in veterinary communication. The veterinary curriculum should include a component on communication training that can help veterinary students acquire necessary communication skills that allow them to effectively communicate with clients and other professionals

**Conclusion:**

This review detailed the complexity of agendas in the field of veterinary communication. The results indicate that veterinary practitioners can further benefit from training on specific communication skills that address the agendas found in veterinary communication research. Furthermore, the veterinary curriculum should include a component on communication training that equips veterinary students with the necessary communication skills that allow them to effectively communicate with different stakeholders such as clients and colleagues with and across the field of veterinary science.

## Background

Communication has always been an important pillar for veterinarians [1]. The ability to communicate effectively leads to better clinical outcomes, such as client satisfaction during the veterinary visit and increased client compliance with the veterinarian’s recommendations [2]. Many factors are known to drive the quality of client–veterinarian communication such as the veterinarians’ communication skills and clients’ expectations [3]. A ‘client-centred’ approach has been promoted to facilitate clients’ adherence, aiming to make more clients decide upon a treatment option in line with the veterinarian’s recommendations. Failure to effectively communicate with clients may result in health, safety and legal repercussions for veterinarians [4, 5]. The quality of communication has a direct impact on the quality of care [1, 6]. In particular, in the field of veterinary communication, there is a growing interest in 1) the ways of delivering difficult news to clients [7, 8], 2) the role of communication skills in the veterinary education curriculum [9–11] and 3) the application of client-centred communication approach within the veterinarian–client relationship [12–14].

Researchers have suggested that a systematic approach of teaching effective communication skills should be included in the veterinary education curriculum. The importance of communication skill education has been highlighted by Haldane et al. [15], whose study indicates that both veterinary practitioners and students ranked verbal communication and interpersonal skills as the most vital abilities for potential veterinary practitioners hoping to join the industry. Moreover, in English speaking countries, the Calgary–Cambridge model is adopted in all veterinary institutions to strengthen the communication skills of the students and consequently improve the outcomes for clients [16]. Psychology courses that include effective communication skills to interact with clients and help them handle bereavement issues have also been incorporated in some veterinary programmes [17]. However, many important communication topics remain missing from the existing veterinary education curriculum, such as ways to provide social support to clients who have lost their pets [18]. Additionally, recent research reveals that communication training in veterinary education is lacking, especially in content-heavy programmes [15], and has revealed that some veterinary students and practitioners possess inadequate communication skills in clinical encounters. Multiple studies have also stressed the need for post-educational training to enhance veterinary practitioners’ clinical communication skills [13, 19, 20].

How veterinarians deliver difficult news to their clients is one of the most researched areas in this field [7, 8]. As summarised by Nickels and Feeley [21], the issues that require communication of bad news to clients in veterinary medicine include 1) pets’ chronic or terminal illness, 2) treatment or treatment failure, 3) unexpected outcomes during the surgery, 4) emergency cases, 5) euthanasia discussions and 6) other potential medical situations. While delivering difficult news, veterinarians often adopt several strategies to ensure the psychological well-being of their clients, such as a careful use of language, the use of open-ended questions, non-verbal skills, relational strategies, developing rapport and empathy with clients, sharing their own experience, respecting the autonomy of clients and avoiding implications of guilt [21]. If veterinarians use such a client-centred approach to communication in extensive discussions with clients, they could better understand the clients’ decisions and address their expectations regarding the care of their pet [16, 22].

Although several studies have recently focused on the above-mentioned three areas in the field of veterinary communication, there seems to be a knowledge gap in this field, as limited studies have taken a holistic approach to study the role of communication in veterinary practices and explore the interactions between veterinarians, clients and other related professionals. To fill in this gap, this integrated review aims to answer the following research questions:

(1) What are the characteristics of veterinary communication reported in these studies?

(2) What are the major findings on veterinary communication?

## Methods

### Search strategy

**Fig. 1:**
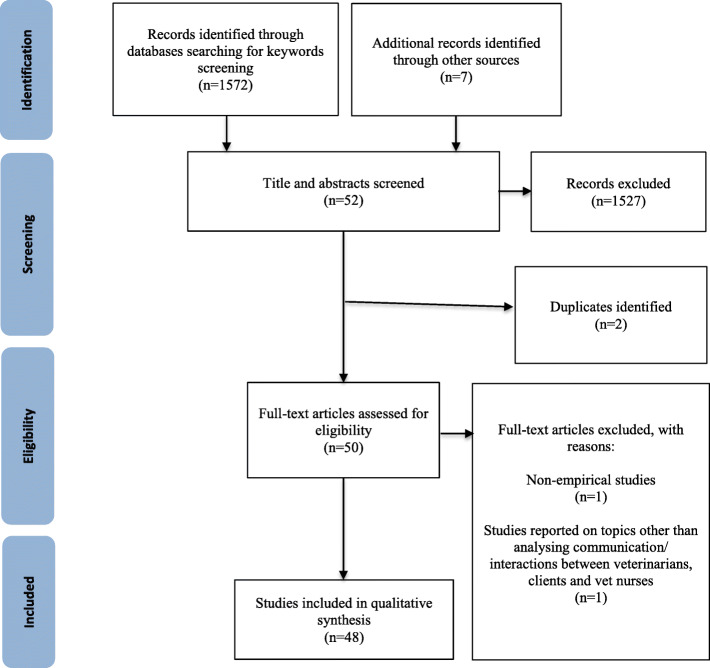
Integrated review of the study

The PRISMA model, an evidence-based minimum set of items for reporting in systematic reviews and meta-analyses, was applied for reporting this review [23] (see Fig. 1). Our search revealed an upsurge in the studies on veterinary communication after 2000; therefore, we focused our search on veterinary communication studies published between 1 January 2000 and 31 December 2018. After discussing with veterinarians and a group of researchers in veterinary science, keywords such as ‘veterinary’, ‘vet’, ‘communication’ and ‘interaction’ were selected as search terms to identify relevant articles.

### Inclusion and exclusion criteria

All included secondary research articles peer-reviewed and selected based on relevant studies related to the topic of the role of communication in veterinary practices in English-speaking countries. After screening the relevant articles, the following three recurring themes were identified and coded by two independent reviewers (the author and a research assistant): (a) client–veterinarian interaction, (b) cross-disciplinary communication in a veterinarian professional team and (c) training of veterinarian communication skills. The following articles were excluded: (a) studies focusing on topics unrelated to veterinary medicine (e.g. the role of pets in human medicine), (b) descriptive studies without the support of any empirical evidence, (c) non-English language articles and (d) non-peer-reviewed studies.

### Selection process

The ERIC, PubMed and PsychInfo databases were searched in the initial screening process to identify relevant studies using the following search terms: ‘veterinary’ or ‘vet’, ‘communication’ and ‘interaction’. Studies that met the inclusion criteria were included for review, and duplicate articles were removed. In addition, a hand search was performed for related studies, and the bibliographies of the included articles were also checked for possible relevant studies. The title, abstract and key information of each included article were reviewed independently by the two reviewers based on inclusion criteria. Each study was then examined by the author and a research assistant. Disagreements were resolved by discussion between the two reviewers. If disagreement persisted, a third person who was a veterinary researcher was consulted to make the final decision. The inter-rater agreement was expressed using Cohen’s kappa coefficient.

### Quality assessment

The quality of the included studies was assessed separately by the author, one research assistant and a group of students with a veterinary background to attain an agreement between the author and other researchers. The Critical Appraisal Skills Programme (CASP) [24–27], a series of standardised and validated checklists that are commonly adopted by scholars in the field of health communication, was used to evaluate the quality of the included studies [28].

### Analysis

A two-step coding approach was applied in the review process. All included articles were read three times to allow the reviewers to familiarise themselves with the results of each study. First, the author read the abstract of each article and decided which articles to include based on the inclusion criteria. Based on the key information from the abstracts and scanning the full texts, the objectives, participant characteristics, study design, method(s) and key findings of each study were summarised (Table 2 in Appendix). Second, each study was examined by the author and a research assistant, and their findings were coded independently according to the research questions for this review analysis: 1) What are the characteristics of veterinary communication reported in these studies? 2) What are the major findings on veterinary communication?

To address these research questions, a subset of coding questions was developed for coding the data into themes, including Participants, Context, Purpose, Method, Data collection and Types of analysis. These themes were checked, repeated, compared and organised for their thematic connections between the annotated notes of the recurring themes. The author and research assistant constantly compared the annotated notes of each study with the rest of the included studies to identify the emerging topics. Subsequently, the second researcher checked and reviewed the included articles.

## Results

### Included articles

The initial screening revealed 1572 related article titles based on our keyword searches, 1524 of which were excluded after reviewing the abstracts as they did not fulfil the inclusion criteria. The resulting 48 articles were subjected to further in-depth review (see Fig. 1). In addition, the following procedures were implemented for ensuring quality of the included studies: screening using keywords, screening the titles, reviewing abstract details, examining the full text and extracting data. For in-depth data extraction, the author and research assistant read the articles in depth and independently filled in a data extraction form [24–28].

For all of the included studies (*n* = 48), the responses to the five evaluative questions were ‘yes’. Similarly, in most of the included studies, ‘yes’ was the answer to at least eight questions from the CASP checklists. This indicates that the included studies were of good quality. No included study was removed because of poor quality. To understand the studies’ contribution to the topic of veterinary communication and the weight of evidence (WOE) of the studies, we also developed an in-depth account of the 48 studies, and only those that met the inclusion criteria have been described and justified below. The account aimed to address the differences in the rating of quality assurance evaluation (i.e. High, Medium or Low) between studies, as well as in each study’s contribution to the review questions, in terms of the following aspects of WOE: 1) relevance of the focus of the study; 2) appropriateness of the study’s research design for addressing the review questions; 3) trustworthiness of the study’s overall methodology; and 4) contribution of the study (as a result of the previous 1–3) to the review questions.

Both the author and research team then checked the completed in-depth review to resolve any identified dissimilarities. The quality evaluation was performed based on each study’s contribution to the review questions (see ‘WOE’ below). The inter-rater reliability of the data extracted by the two independent reviewers was calculated. The result of quality judgement was high, with less than 2.0% disagreement.

### The weight of evidence (WOE)

The WOE was measured based on the following four aspects: (1) the relevance of each study to the review; (2) the appropriateness of the research design; (3) the trustworthiness of the reported research findings; and (4) the contribution of the study to this review. Despite the degree of subjectivity in the process of WOE measurement, a fairly representative picture of the overall research in veterinary communication can be observed in Table 1. Among the included articles, 83% (*n* = 40) were found to have high or medium relevance to this review and 46% (*n* = 22) used research designs that were considered as highly appropriate in addressing the review questions. Furthermore, 27% (*n* = 13) of the articles showed a powerful contribution in addressing the review questions and 56% (*n* = 27) showed a fair contribution. These results indicate that regardless of the increasing research interest in communication in veterinary science, more future studies with a rigorous research methodology are warranted.

**Table 1:** Weight of evidence of the research articles

	Relevance n (%)	Appropriateness of research design n (%)	Trustworthiness n (%)	Overall contribution n (%)
**Low**	8 (17%)	5 (10%)	5 (10%)	8 (16%)
**Medium**	23 (48%)	21 (44%)	29 (60%)	27 (56%)
**High**	17 (35%)	22 (46%)	14 (30%)	13 (27%)

### The included studies

The details of the 48 studies that fulfilled the inclusion criteria are summarised in Table 2 in Appendix. The number of published articles on veterinary communication increased between 2007 and 2017. Most of the included studies were found in the United States (*n* = 19), the United Kingdom (*n* = 14), Canada (*n* = 4), Australia (*n* = 3), and other European countries (*n* = 8) such as the Netherlands (n = 3). This finding can be explained by the number of representative veterinary-related professions and universities with veterinary colleges in these countries. In terms of the methodology, qualitative interviews and quantitative surveys were the most common tools used in the studies to record the personal experiences of veterinarians and assess their communication skills [56].

### Themes of the included studies

As thematic analysis can be adapted to the aims of the research, instead of using a set of pre-assigned themes in this review, the author sorted, identified and explored the thematical relationships of the coded issues from each study, which were categorised into the following broader categories: 1) client–veterinarian communication; 2) cross-disciplinary communication in a professional veterinarian team; 3) training of veterinary communication skills. Categorised themes were subjected to the common steps of thematic analysis (familiarisation, coding, generating themes, reviewing themes, defining and naming themes, and writing up) [57].

### Client–veterinarian communication

Client–veterinarian communication was found to be the most recurring theme in the selected studies. Clinical communication skills were mostly investigated by comparison between human medicine [9, 58]. The client-veterinary interaction is delineated in an initial greeting, history taking, performing physical examination, explaining diagnosis, offering treatment options and closing the interaction. Trust and rapport are built up throughout the process through the veterinarian’s usage of communication strategies and interpersonal skills.

Although several steps of consultation are common between veterinary and human medical consultations (e.g. treatment plans), fundamental differences exist between them. One is that pets cannot verbalise their medical concerns to the veterinarian, and thus, the veterinarian relies on the owners’ explanations or clinical examinations to determine the pet’s illness. Admittedly, this is a situation comparable to paediatrician-patient communication, as young children are also not able to communicate their concerns clearly, rendering healthcare practitioners dependent on communication with caretakers [59, 60]. Therefore, development of trust in a client–veterinarian relationship is crucial as it will not only enhance the quality of history taking but also will allow better clinical diagnosis and subsequent treatment care.

Further, several studies have emphasised the constraints of insufficient consultation time [11, 12, 31]. On average, veterinarians spend only 24 min per case [11], and many veterinarians have to deliver all the necessary information within this time. This prompts some clients to search for further veterinary information online, which could be misleading and incorrect [12]. To prevent clients from receiving unreliable information, veterinarians should educate clients about ways to identify reliable sources of information online [12]. For example, the overuse of antibiotics is a real concern for many veterinarians. Despite increasing awareness about the development of resistance due to antibiotic overuse, most clients still have misconceptions about the appropriate use of antibiotics [39]. Another challenge faced by many veterinarians is that some clients ignore the veterinarian’s recommendations. If the clients do not adhere to the professional advice and prescribed diet, the results could be life-threatening for the pet. One study suggested that clients with a closer relationship with their veterinarian were more willing to follow their veterinarian’s suggestions and pay for their pet’s treatments because they understand the recommendations well [6]. This can be achieved with long-term trust between the clients and their veterinarian. One approach to develop trust is to share personal experiences and inform all possible options. Clients can then feel assisted instead of being forced to make certain decisions [21]. When the veterinarians show their willingness to help, the clients are more likely to select the recommended treatment options. Another approach is to avoid misunderstanding; the veterinarians can spend more time with their clients to ensure that the clients understand the explanations. Such a careful approach would help build a trustful and reliable relationship between the two parties [12]. Absence of trust in any client–veterinarian relationship can lead to miscommunication and no adherence to treatments, which is likely to reduce the client’s satisfaction and put animal safety at risk [41].

The importance of nonverbal communication within client-veterinarian communication should also be acknowledged. Sutherland [61] writes that these nonverbal cues during a conversation may be far more important than the verbal content, as nonverbal communication can alter the meaning of a statement. As such, it is important for veterinary practitioners to develop the ability to pick up nonverbal cues from patients and alter their actions accordingly as this skill will prove useful in various contexts, such as when the veterinarians need to respond to clients’ emotions. For example, the veterinarians can maintain good eye contact, spend time with the client and listen to their needs, all of which can improve history taking and consequently the prescription of a more comprehensive care plan and recommendations [1, 21]. Clients’ negative emotions can also be identified by veterinarians who understand subtle cues or overtly shared verbal concerns. Veterinarians can also use nonverbal communication for delivering a message to the client, utilizing facial expressions and body language to promptly provide emotional support using a mix of appropriate and effective verbal and non-verbal communication [33]. Veterinarians should consider four forms of non-verbal cues while communicating with clients: kinesics, proxemics, paralanguage and autonomic shifts. Kinesics includes facial expressions, level of body tension, touch and movement, whereas proxemics is related to the shaping of space between the client, animal and veterinarian. Paralanguage involves voice-related components such as pitch, tone and volume. Autonomic shifts are an unpredictable variable as they are governed by the autonomic nervous system [40].

Delivering difficult news is considered as the most important aspect of communication for many veterinarians [14]. The strong relationship between clients and their pets makes it challenging for many clients to accept any difficult news as they view their pets as their family members [11]. When delivering difficult news, veterinarians are required to attend to the client’s emotional needs using communication strategies to minimise their negative experiences or risk of trauma [44, 45]. Delivering difficult news can be especially challenging when the veterinarians have to communicate with vulnerable groups [44]. Modified communication strategies can be used to fulfil the expectations of the vulnerable groups, such as children and the elderly. For children, using specific ways of delivering bad news is important to reduce trauma at the developmental stage. When their pets are ill or could die, it is better to first communicate with their parents about ways to share the bad news with their children, and following an honest, simple and kind approach is recommended [44]. Likewise, when attending to the elderly, communication should focus on their connection with the pet. According to Bateman, communication skills are crucial for dealing with the situation without causing additional problems [45]. When the veterinarians can skilfully present bad news, they need not behave defensively during a difficult conversation, which allows time for their clients to accept and understand the information. It is also important to assess the preferences of the clients before delivering the difficult news. There are existing frameworks in human medicine that provide a step-by-step guide on how to break bad news, such as the COMFORT (Communication, Orientation, Mindfulness, Family, Ongoing, Reiterative messages, and Team) model and SPIKES (Setting, Perception, Invitation, Knowledge, Empathetic Response, Summary) protocol. However, research into developing a specific model for delivering difficult news in veterinary medicine has been limited. Veterinarians also face tremendous pressure while delivering bad news, which has been reported as one of the common factors of occupational stress in the veterinary career [30]. Thus, it is important to teach useful communication skills in the veterinary curriculum that can help veterinary students deliver bad news and avoid any stress due to miscommunication during the veterinary clinical practice. There are different approaches to minimise the stress caused to both the veterinarians and clients when delivering/receiving bad news [35]. As the loss of a companion animal can be traumatic and occasionally very difficult to accept for many people, veterinarians can invite clients to visit with a family member or friend as he/she could provide the necessary support to the clients when they receive the bad news [7, 34]. There are many ways to express empathy. For example, a verbal condolence can provide emotional support, in addition to other approaches such as a sympathy card, informational support (i.e. grief management hotlines, brochures and booklets) and supporting non-verbal communication (e.g. hand on shoulder and back or hug) [21]. The level of support offered by the veterinarians can eventually help develop a trustful and sustainable relationship between the clients and their veterinarian [21]. Further, when explaining the diagnosis, information should be provided to the clients in a series of chunks. The veterinarians can provide signposting before delivering the message directly in explaining a diagnosis to a client [21]. Another approach is to keep the message simple and ensure that the clients understand the conditions completely [21]. Once a range of possible treatments options are provided and their potential risks are explained, clients can take an active role in making an informed decision that addresses their expectations [7]. Another factor to consider while delivering bad news is the setting. For example, a private room without distractions, and the availability of private exits, have frequently been suggested in several studies for creating a peaceful and calm atmosphere [21]. Considering animal welfare based on the diagnosis, euthanasia can be the most suitable option when the pet’s quality of life is seriously impaired [7]. Clients can be better informed about the worst scenario when they realise the seriousness of the situation. Although euthanasia is a hard decision to accept, clients with a close relationship with their veterinarian are more likely to accept it as an alternative while minimising guilt and additional financial burden [21].

Clients’ understanding and treatment adherence are the direct emotional rewards derived by building a trustful client–veterinarian relationship. The first consultation is critical for the veterinarians to make a positive professional impression on their clients, which can ultimately affect a client’s decision of returning or switching to another clinic. Furthermore, veterinarians can use special interactional strategies such as baby-talk for building the interpersonal relationship [29, 31]. Without mutual trust, clients will eventually move to another veterinarian. To build a sustainable relationship, veterinarians can adopt suitable behaviours to present a reliable and competent image to their clients. For example, they can present their clinical diagnosis in a professional manner by explaining the diagnosis concisely and clearly and offer possible treatment options in a simple and comprehensible manner. Veterinarians should be patient and confident and show interest in the treatment and welfare of the animals, which can allow the development of a better client–veterinarian relationship [34]. The use of language should be kept relatively simple by avoiding jargon to ensure that the clients understand the information [7, 21]. This is especially important in cases related to euthanasia. A soft tone of voice is also suggested to keep the atmosphere peaceful [21].

In the farm animal production context, the client–veterinarian communication is different because the major focus is on the health of the animal group rather than individual well-being. Veterinarians generally focus on the big picture in cases of farm animals. If the veterinarians do not use suitable communication strategies, the clients may misunderstand information on life-threatening conditions, which may lead to a large-scale zoonosis crisis [19]. Another possible reason for such outcomes is that farmers and veterinarians tend to focus on different concerns [36]. Famers generally struggle with financial constraints as they are running a business and tend to focus on minimising costs and short-term repercussions [50]. Moreover, when there are suspected cases of diseased animals, farmers can withhold from reporting to the veterinarian [19]. However, from a veterinarians’ perspective, the public health and wellbeing are a priority. In this case, disagreements can occur and the relationship between the two parties can be threatened. The unwillingness to adhere to recommendations can reflect distrust in the client–veterinarian relationship. Finally, to manage emerging zoonotic diseases, veterinarians should promptly provide necessary knowledge and communicate about emergency measures. When farmers understand the severity and risks of the possible zoonotic outbreak, they are more likely to follow professional advice. In addition, the veterinarians can allow the farmers to acquire an evidence-based understanding of the situation and corresponding actions [16]. It is also important for the veterinarians to explore the farmers’ motivation and understand their values and goals, which can be achieved by involving the farmers in the treatment plans and developing feasible solutions to enhance the client–veterinarian relationship [16].

### Cross-disciplinary communication in a veterinarian professional team

In addition to the importance of the client–veterinarian communication, the veterinary profession necessitates teamwork. Occasionally, communication problems can occur between the veterinarians and other clinic staff. Ineffective communication is a major cause of critical incidents, which may result in animal harm and death and is the most common cause of complaints. For example, the lack of communication between the receptionist and veterinary surgeon has been reported as a major cause of communication errors [5]. Most surgeons value autonomy, but individualism and autonomy are not suitable or beneficial behavioural approaches within a group of professionals [5]. In most cases, there is limited time for communication, and team communication is problematic and lacks structure, resulting in possible miscommunication and clinical errors.

Communication issues within the veterinary team have been emphasised in the study by Ruby and DeBowes [48]. These researchers found that team communication in veterinary practice is challenging. Although training on team communication is included in the veterinary curriculum to prepare the students to become professional team players, several key elements are missing. To develop an effective team, one needs to decide consciously, display transparent governance, set clear expectations of the teammates, and avoid making assumptions. It is also important to motivate the rest of the team to be responsible for adapting to the agreed standards and implement them in daily clinical practice. The team leader can share important values to shape the team identity while maintaining a supportive and positive working climate.

Another important requirement is the ability of the team to develop conflict-resolving strategies, such as setting regular meetings to let all members participate in reaction and response to each other, and providing feedback that promotes self-reflection.

### Training on veterinary communication skills

The importance of communication skills has been emphasised in all included studies. In the study by McDermott et al. [13], 98% of respondents believed that communication skills are equally important to or more important than practical clinical skills. Training on communication skills in the veterinary curriculum is very limited [13, 37]. The existing veterinary curriculum does not fully address the needs in this aspect of practical skills. In one study, less than 50% of the participating veterinarians had received communication skills training in veterinary schools, 65% believed that the veterinary school did not fully prepare them to face communication issues in their career, and 50% had to attend post-graduate communication training workshops [13]. The continuous professional education currently available mainly includes simulated consultations and online training, offering less time investment while attending to money and applicability considerations.

Some veterinarians believe that learning communication skills from real-life cases throughout the veterinary career is more effective and applicable [13]. The communication skills that most veterinarians develop vastly depend on their everyday experiences. Although self-development through real-life experiences is critical for developing a unique yet suitable approach to communication, students should receive professional guidance for developing specific competence to communicate effectively in their early career [7].

There has been an upsurge in studies emphasising communication education. Organisations such as the Veterinary Defence Society have been sponsoring communication skill training courses for undergraduate veterinary students in the UK and Ireland for several years [7]. Generally, communication courses are spread throughout the whole curriculum to allow learning when students are on clinical rotation [49]. On average, only 15 h of discussion time are allocated for end-of-life topics in the intensively scheduled veterinary school curriculum [19].

Studies have also examined the contexts and scenarios that veterinary students are recommended to face during their communication skill education. According to Von Fragstein et al.’s consensus statement on medical school communication, which aimed to help medical schools provide an appropriate mix of communication learning experiences for students, students were recommended to engage with scenarios such as end-of-life communication, navigating cultural and social areas of sensitivity, dealing with communication impairment, and communicating about emotional topics. Studies have explored different teaching approaches or interventions for delivering veterinary communication skills, including lecture-based teaching, role play interactions, small-group discussions and real-life application [10]. Furthermore, studies have emphasised the importance of ethical considerations, especially in the topics of end-of-life conversation or euthanasia. Specific communication protocols have also been established for training in delivery of bad news. For example, the COMFORT (communication, orientation and opportunity, mindfulness, family, opening, relating and team) model is also applicable in veterinary medicine and training sessions as it considers different aspects of social support [21]. The model addresses cost-related issues and euthanasia, which are not found in human medicine practices [20]. This consultation model emphasises the importance of a client-centred approach. Veterinarians are recommended to encourage participation, negotiation and knowledge sharing instead of one-directional information provision [11]. This approach can create a welcoming setting and thus give a positive experience to the clients.

In general, lecture-based discussion about euthanasia occurs in the early years of veterinary school [50]. Kolb’s experiential learning theory is already a part of the veterinary curriculum, and one study showed that evaluation of real-life recorded consultations can be a reflective way of communication education [54, 62]. In addition, simulations can enhance the students’ ability to develop empathy by increasing their ability to understand the clients’ feelings and emotions [49]. Two studies have reported the advantage of the shadowing approach, stating that the coaching process allows self-development by observation [11, 16]. The unique benefit of this learning approach is that every student can have his/her own unique experiences.

In addition to the traditional teaching approaches, several innovative approaches to delivering communication using technology can be applied. For example, web-based learning is expected to become a major learning approach in veterinary medicine [2] and is already a valid and reliable communication teaching method in human medicine. Although no study has yet reported the same benefits of web-based learning in veterinary medicine, this approach is expected to have the same potential in veterinary communication education. Use of virtual patients is considered as an effective way to assess students’ communication skills in human medicine and as a potential communication assessment tool in veterinary medicine [2].

Although most studies have suggested changes in communication skills training, some limitations still remain in group training. For example, group training might compromise individual needs [55]. Hence, the idea of tailor-made training has been introduced, but it was not concluded to be a practical strategy in large group training [55]. One study suggested that when graduated students start practicing veterinary medicine, providing more continuous professional training (CPT) can reduce the communication skill gaps between senior veterinarians who graduated before 2000 and current veterinarians [13]. However, there is a lack of studies focusing on CPT for farm animal veterinarians [36], suggesting a lack of emphasis on communication skills of large-animal veterinarians.

## Discussion

This integrated review of the role of communication in veterinary clinical practice highlights the significance of client–veterinarian communication and the lack of communication training in the veterinary curriculum. Most of the included studies that have investigated the nature of veterinarians’ communication with their clients have emphasised that it not only affects the relationship between clients and the veterinarians but also the health of the animals. The review showed that the interaction between the clients and the veterinarians could be challenging as various types of dilemmas occur during different stages, such as insufficient consultation time, challenges in delivering difficult news, misconception and misunderstanding, and poor treatment adherence, echoing the findings of a review conducted by Cornell and Kopcha [63]. Despite the importance of maintaining effective communication and sufficient information exchange for different stakeholders throughout the diagnosis and treatment of animals, there is a lack of appropriate measures to tackle issues in these matters. The review supports the findings of [15, 18], and shows that systematic communication education is absent in the existing veterinary curriculum. Although studies such as McDermott’s [13] and Jackson’s [7] have explored the possible future approaches, it is obvious that the current curriculum is not yet well developed and adequate, likely resulting in communication challenges for veterinarians in their practice. Consistent with a previous study that stated that interprofessional working is under-theorised and under-researched [5], this review highlights the lack of studies in the context of communication between veterinary professionals. Similar to the setting of human medicine, the approach and content of communication between the doctors or veterinarians and the nurses and receptionists may have a huge impact on the clients’ impression of the veterinarians and their perception of their pets’ healthcare. This is a finding that is similar to the results of a study conducted by Coe et al. [64]. However, not many studies have focused on this aspect of communication between the professionals or that between the farmers and veterinarians. Notably, a large body of evidence is available on the aspects of communication between the veterinarians and the clients and the associated challenges; however, more systematic research is needed to obtain a more comprehensive understanding of the communication between the veterinarians and other stakeholders, such as receptionists and clinical staff members.

This review has a few limitations. Although the findings were derived from heterogeneously designed studies, only studies published in English were included. As a result, the geographic focus of this research was also mainly on Western countries. Studies published in local languages that may reflect more detailed aspects of interpersonal veterinary communication affected by cultural factors were not included. Thus, the findings may be biased in terms of the overall social aspects. A systematic approach comparing diverse studies to understand the cultural differences between Eastern and Western countries would be meaningful. Thus, more in-depth studies examining how different cultures adopt distinct approaches to veterinary communication and influence the surrounding environment are warranted.

## Conclusion

This review identified a gap in the communication skills of veterinarian professionals as well as a communication gap between different veterinarian professionals and the outcomes of effective client–veterinarian communication. For animals, effective communication between their owners and the veterinarians will allow better medical care in terms of drug route, dose and frequency and annual health check; better home routine care such as diet, exercise and wound care; and, consequently, an increase in their overall quality of life. Adequate and clear communication during veterinary consultations would allow clients to acquire the correct information about pet care and facilitate the history taking of pets by veterinarians, which could increase the clients’ satisfaction with the clinic experience and reduce stress [36]. Finally, from the perspective of veterinarians, effective communication can allow them to properly care for and treat the pets and avoid stress, foster a positive working environment, and enhance the effectiveness and efficiency of teamwork. The reviewed studies confirmed the importance of and the roles of communication in veterinary medicine and the need for a more comprehensive curriculum for teaching veterinary communication skills. This review highlights the requirement of more research to explore the culturally influenced communication approaches that veterinarians in non-English speaking countries adopt so as to develop more effective communication models than those commonly used in veterinary practices in Western countries.

## Data Availability

The datasets used and/or analysed during the current study are available from the corresponding author on reasonable request.

## Appendix

**Table 2:** Themes emerged from the included studies

Sub-themes	Key findings	Studies
(1) Client-veterinarian communication	The facilitation of utterances that address the animals directly or on behalf them can promote a healthy client-veterinarian relationship.	[29]
	Gaining a better understanding of the pets’ dietary histories through routine communication allows veterinarians to offer more effective health care recommendations to the clients.	[1]
	Although poor client-veterinarian relationship is one of the sources of occupational stress in veterinary practices, veterinarians still find themselves enjoying the career because of the human-animal bond and the satisfaction of improving animal welfare.	[30]
	To ease the process of delivering bad news, it is important to deliver information little by little, keep the language simple and clear, provide every possible treatment with related information and give sympathy to the clients.	[7]
	Nigel Hough received several charges due to inadequate overnight care for a Staffordshire bull terrier, miscommunication with the clients and using disparaging language to the clients.	[31]
	In both cases of small animal practices and large animal production, thinking in the clients’ perspectives and understanding their own situations and constraints is a key to build a healthy relationship.	[32]
	The VR-CoDES model for analyzing clients’ negative emotions in human medicine is applicable in veterinary medicine, although limitations exist since there are extra considerations in veterinary medicine such as guilt, reassurance and cost discussion.	[33]
	The stressor of client-veterinarian relationship is usually the time when euthanasia is the most suitable decision for the animals or the sudden loss of the animal.	[34]
	The communication models SPIKES and COMFORT in human medicine are largely applicable in veterinary medicine. However, both models do not fully accounted every strategy reported by veterinarians. Currently, COMFORT would be a more preferable model to use in veterinary medicine.	[21]
	Effective communication can build a close client-veterinarian relationship, which further improves animal care.	[6]
	Veterinarians rated delivering bad news as moderately stressful and the stress level usually declines gradually when they experience more bad situations.	[35]
	Majority of veterinary students believe there should be more emphasis on end-of-life issues in order to fully prepare them to face terminally-ill animals.	[36]
	In terms of emerging zoonosis risks in large animals workplace, veterinarians should receive more training on discussing risks and management strategies with farmers in order to ensure public health and human safety.	[3]
	Veterinary students feel unprepared to bring bad news to clients and therefore they seek for more communication skill training with stimulated pet owners.	[37]
	Reassuring clients with empathetic communication gives clients a higher satisfaction in the clinic visit experiences.	[38]
	Majority of veterinarians in United Kingdom think that clients obtain online information about pet health and most of them cannot fully understand the information given, therefore resulting in a negative impact on animal health, as well as on the client-veterinarian relationship.	[12]
	There are misunderstanding and misconceptions about the use of antibiotics between clients and veterinarians, therefore leading to antimicrobial resistance.	[39]
	A mutualistic and paternalistic approach in client-veterinarian relationship is more preferable in the large animal production context.	[16]
	The mnemonic tool ADOBE is useful for veterinarians to bring the clients from the challenge zone to the comfort zone in order to create a comfortable environment and to facilitate client-veterinarian communication.	[14]
	Nonverbal behavior can help create a safe space for the clients to share information about their pets and to show their emotions. This set of skill can be applied everywhere and can most definitely create a satisfied partnership for the client and veterinarian.	[40]
	Improved communication with pet owners is proved to be associated with fewer complaints and higher level of satisfaction. The emerging acknowledgement of the importance of communication and relationship building contributes to the growing recognition of the skills; significance.	[4]
	Veterinarians may face different opinions with clients due to ethical dilemma but they should be comfortable in drawing boundaries and explaining the underlying values with their clients. Ethical issues and morally problematic situations should be acknowledged in order to improve veterinarians’ practice and to provide a high level of care for pets.	[41]
	Both clients and veterinarians should accept the inherent risks in diagnostic and treatment decisions. Situations that deal with client disappointment have to manage carefully. Responsibility should be accepted, while acting ethically, sensitively and trying to reach the best solution possible. The effort of addressing disappointment and maximizing client satisfaction can help build rapport and loyalty among customers.	[42]
	Skillful end-of-life discussions are important for veterinarians and it is seen as a core clinical skill, as well as an integral part of the job. Facilitating a good discussion can help ease the emotional distress of the clients and maintain a long term relationship and enhance veterinarian and client satisfaction.	[8]
	Treating clients in a respectable manner and acknowledging the information as well as animals’ diagnosis is about them can help veterinarians preserve relationship with their clients.	[43]
	The stress level of veterinarians and their clients when breaking bad news is significantly different. There is no evidence that following suggestions of how to deliver bad news have effectively lower the stress experienced by the receiver. Giving bad news gets easier with practice as veterinarians will be less likely to get caught off guard by a client’s response.	[35]
	There is a lack of training in veterinary school on how to communicate social support to grieving clients. Most veterinarians learned through experiences and they believe such training will be beneficial as these are stressful situations that are highly common during practice.	[18]
	The potential benefits of introducing an interdisciplinary field of veterinary health psychology is highly promising as it can help veterinarians deal with cope, decision making and end-of-life matters. A program should be devoted to understand how psychology affects and benefit veterinary medicine.	[17]
	There is a need for more research on low-cost and community-based veterinary medicine. More research is needed to improve animal care and animal welfare in low-income communities, and provide low-cost clinics training in school curriculums for the underserved populations.	[22]
	Veterinarians cannot avoid encountering clients of all phases of life span and it is essential to communicate with them in a compassionate, respectful and honest communication. It can help improve clients veterinarians relationship and advance the field of veterinary medicine.	[44]
	Bad news should be delivered skillfully by using various strategies to ensure a better experience for the clients and be more acceptable to the outcomes. Veterinarians can benefit from learning the skills and techniques used in emergency settings to make clients more comfortable when receiving bad news.	[45]
(2) Cross-disciplinary communication in a veterinarian professional team	Under the high pressure and high workload working environment in the veterinary clinic, verbal and written communication become critical to reduce communication errors that are usually evitable with effective communication between receptionists, veterinary nurses and veterinary surgeons.	[5]
	Social media acts as an important platform for international knowledge sharing in order to exchange experiences between geographically different veterinarians in the context of veterinary practices.	[46]
	Veterinarians, technicians and shelter workers are highly at risks of having compassion fatigue as they care very deeply for animals that they often fail to care for themselves. Self-care and overcoming compassion fatigue can be rewarding and can lead to a healthier balance between life and work.	[47]
	Teamwork is highly significant in the practice of veterinary medicine. Though there are challenges in creating a good relationship among the members, but efforts like weekly meetings, committing to their jobs and showcasing trust can come a long way in creating a better environment for the practice.	[48]
(3) training of veterinary communication skills	The expected outcomes through communication training with real life interpersonal challenges are developing empathy and ethical awareness.	[49]
	Problem-based communication training approach prepares veterinary students to develop a client-centered communication skill in order to enhance client-veterinarian relationship.	[11]
	Addressing veterinary students’ understanding of euthanasia, death and illness in earlier stage of the veterinary school curriculum can better prepare them for facing emotionally difficult situations in the clinical placements, as well as future practices.	[50]
	Veterinary students appreciate learning communication skills through experimental learning experiences, where they observe real life cases during clinical rotations.	[51]
	There are increasing emphasis on communication skill training in veterinary schools in United Kingdom, where different approaches are planned throughout the curricula.	[10]
	Most of the communication related encounters are the same for both veterinary medicine and human medicine, but the major differences between two are the additional communication requirements in veterinary medicine, where veterinarians need to deal with discussing financial issues, treatment options and euthanasia.	[20]
	Workshops based on translational research that focuses on the flow of communication between farmers and veterinarians can ease the communication and therefore leading to a better dairy farm management.	[36]
	The concept of ‘One Communication’ should be implemented in the curricula of veterinary medicine in order to facilitate communication between different stakeholders in the perspectives of small animal practices, farm animal production, infectious disease, public health and pharmaceuticals.	[52]
	Web-based learning teaching in communication skills has shown its reliability and validity in human medicine and therefore similarly, it can be a future direction for communication education in veterinary medicine.	[53]
	Kolb’s Experiential Learning Theory (ELT) was tested to be a useful framework in communication education in veterinary medicine.	[54]
	Veterinary communication skill is one of the core competencies and should be prioritized from the early veterinary school curriculum.	[55]
	Web-based learning is considered effective for communication education especially to access communication skills in human medicine and it will be a possible approach in veterinary medicine.	[2]
	Nearly 100% of the respondents believe that communication skills are as important as or more important than clinical skills and 40% are interested in further simulated online training.	[13]
	Veterinarians have strong desire to help and bring comfort to the clients when euthanizing animals, they need to show understanding and personal treatment during the procedures. They recognize client-present euthanasia is power and a grief-intervention tool, it can effectively build client veterinarian relationship. With experience, veterinarians develop the ability to serve as grief counselors though they are not trained as one. The majority of the respondents think veterinarian training had not prepared them well enough to handle and communicate with owners of terminally ill animals. An overwhelmingly large amount agree that schools should place more emphasis on communication skills with owners of animals that had to go through euthanasia.	[19]

## Abbreviations

CASP: Critical appraisal skills programme
COMFORT: Communication, orientation and opportunity, mindfulness, family, opening, relating and team
CPT: Continuous professional training
PRISMA: Preferred reporting items for systematic reviews and meta-analyses
Vet: Veterinary
WOE: Weight of evidence
